# Supplementary material for: Randomised Controlled Feasibility Study of the MyHealthAvatar-Diabetes Smartphone App for Reducing Prolonged Sitting Time in Type 2 Diabetes Mellitus
Source: Int J Environ Res Public Health. 2020 Jun 19;17(12):4414. doi: 10.3390/ijerph17124414 (PMC7345154; doi:10.3390/ijerph17124414)
Supplement: Supplementary file 1 [file ijerph-17-04414-s001.zip › Supplementary File S1 Intervention text message content.pdf]

File S1: Intervention text message content

Week 1: G.R.O.W

What sitting time goals do you hope to achieve?

Sitting for shorter durations, sitting less throughout the whole day, getting up and moving around more often?

How can you make your goals SMART?

Specific, Measureable, Achievable, Relevant and Timely

Week 2: G.R.O.W

How would sitting less benefit you?

Better quality of life, more mobile, weight loss, better glucose control, something else?

What are the negative outcomes of sitting too much?

Poor health/ fatigue/ weight issues/ low fitness

Week 3: G.R.O.W

What has stopped you from sitting less in the past?

Time, opportunity, work, cost, motivation, something else?

What have you tried before to help you to sit less?

Changing your environment, making plans to sit less, giving yourself cues/reminders to move

Week 4: G.R.O.W

What can you do differently going forward, to help you reach your goal of sitting less?

Assign a certain day or time, set an alarm, get others involved, something else?

When will you know you have reached your intended goal?

When I lose X amount of weight / when I complete X amount of steps per day

Week 5: G.R.O.W

What support is available to you to help you to sit less?

Mobile phone app, internet, diabetes support groups, NHS website, something else?

Who can help you to reach your goal?

Peers / family / friends / colleagues / support groups

Week 6: G.R.O.W

How does it feel when you are meeting your goal?

Positive, happy, energetic

How can you continue to make positive changes?

Make plans, ask friends to help

Week 7: G.R.O.W

On a scale of 0–10, how important is it for you to make changes to help you to sit less?

What made you chose this number?

What are your next steps to maintaining and reaching new goals?

Working with friends, SMART goals, something else?

Week 8: G.R.O.W

What barriers might you have to overcome to reach and maintain your goal?

Work, time, money, transport, motivation, something else?

How will you reward yourself when you complete your intended goal?

Buy a new outfit / book a holiday / take some time to relax / something else?
