# Supplementary material for: Randomised Controlled Feasibility Study of the MyHealthAvatar-Diabetes Smartphone App for Reducing Prolonged Sitting Time in Type 2 Diabetes Mellitus
Source: Int J Environ Res Public Health. 2020 Jun 19;17(12):4414. doi: 10.3390/ijerph17124414 (PMC7345154; doi:10.3390/ijerph17124414)
Supplement: Supplementary file 1 [file ijerph-17-04414-s001.zip › Supplementary table S2 - psychological outcomes.docx]

**Table S2.** Within and between-group differences from baseline to follow-up for psychological outcomes. Data presented as mean (SD).

| **Variable** | **Control baseline** | **Control follow-up** | **Within-group differences** | **Intervention baseline** | **Intervention follow-up** | **Within-group differences** | **Between group differences** | **Cohen’s d** |
| --- | --- | --- | --- | --- | --- | --- | --- | --- |
| Theory of Planned Behaviour |  |  |  |  |  |  |  |  |
| Attitude | 5.2 (2.0) | 5.5 (1.6) | 0.3 (2.2) | 5.4 (0.6) | 6.1 (0.6) | 0.6 (0.8) | 0.4 (2.1) | 0.18 |
| Subjective norms | 4.4 (1.3) | 4.6 (0.8) | 0.2 (1.2) | 4.5 (1.0) | 4.1 (1.1) | -0.4 (1.3) | -0.6 (2.2) | 0.34 |
| Perceived behavioural control | 4.3 (0.6) | 4.9 (0.5) | 0.6 (0.7) | 4.7 (0.4) | 5.0 (0.8) | 0.3 (0.8) | -0.3 (1.1) | 0.40 |
| Intention | 5.3 (1.1) | 5.6 (1.0) | 0.4 (1.1) | 5.2 (1.3) | 6.1 (0.6) | 0.9 (1.6) | 0.5 (2.0) | 0.36 |
| Planning |  |  |  |  |  |  |  |  |
| Sitting Self-efficacy | 14.9 (4.3) | 17.2 (1.9) | 2.3 (4.3) | 15.4 (3.8) | 15.2 (3.3) | -0.2 (3.6) | -2.6 (5.8) | 0.63 |
| Planning | 1.4 (0.6) | 2.1 (0.8) | 0.6 (1.2) | 1.6 (0.9) | 2.7 (0.7) | 1.1 (1.2) | 0.4 (1.7) | 0.42 |
| Wellbeing |  |  |  |  |  |  |  |  |
| WEMWBS | 50.6 (4.8) | 50.6 (6.1) | 0.0 (3.2) | 51.0 (8.1) | 53.2 (8.9) | 2.2 (10.9) | 2.2 (9.5) | 0.27 |
| Positive affect | 35.4 (4.9) | 33.4 (5.8) | -2.0 (3.6) | 32.1 (7.2) | 35.6 (5.7) | 3.4 (9.9) | 5.4 (10.7) | 0.72 |
| Negative affect | 16.1 (4.9) | 15.1 (5.2) | -1.0 (4.6) | 16.6 (6.6) | 14.6 (4.1) | -2.0 (6.6) | -1.0 (8.1) | 0.18 |
| Life satisfaction | 6.4 (2.5) | 6.4 (2.1) | 0.0 (2.5) | 6.9 (1.5) | 7.3(1.7) | 0.4 (2.1) | 0.4 (3.8) | 0.17 |
| Worthwhileness | 8.0 (1.9) | 7.4 (0.7) | -0.6 (2.1) | 7.4 (1.5) | 7.7 (1.3) | 0.2 (1.4) | 0.8 (2.6) | 0.45 |
| Happiness | 7.8 (2.0) | 7.3 (1.6) | -0.4 (2.8) | 7.2 (1.5) | 7.8 (1.3) | 0.6 (2.1) | 1.0 (4.2) | 0.40 |
| Anxiety | 3.0 (2.8) | 2.6 (1.9) | -0.4 (1.6) | 2.78 (2.1) | 2.78 (2.3) | 0.0 (1.6) | 0.4 (2.5) | 0.25 |

Note: WEMWBS—Warwick-Edinburgh Mental Well-being Scale.
