# Supplementary material for: Randomised Controlled Feasibility Study of the MyHealthAvatar-Diabetes Smartphone App for Reducing Prolonged Sitting Time in Type 2 Diabetes Mellitus
Source: Int J Environ Res Public Health. 2020 Jun 19;17(12):4414. doi: 10.3390/ijerph17124414 (PMC7345154; doi:10.3390/ijerph17124414)
Supplement: Supplementary file 1 [file ijerph-17-04414-s001.zip › Supplementary table s2 - psychological outcomes.pdf]

Table S2. Within and between-group differences from baseline to follow-up for psychological outcomes. Data presented as mean (SD).

Variable	Control baseline	Control follow-up	Within-group differences	Intervention baseline	Intervention follow-up	Within-group differences	Between group differences	Cohen's d
Theory of Planned Behaviour								
Attitude	5.2 (2.0)	5.5 (1.6)	0.3 (2.2)	5.4 (0.6)	6.1 (0.6)	0.6 (0.8)	0.4 (2.1)	0.18
Subjective norms	4.4 (1.3)	4.6 (0.8)	0.2 (1.2)	4.5 (1.0)	4.1 (1.1)	-0.4 (1.3)	-0.6 (2.2)	0.34
Perceived behavioural control	4.3 (0.6)	4.9 (0.5)	0.6 (0.7)	4.7 (0.4)	5.0 (0.8)	0.3 (0.8)	-0.3 (1.1)	0.40
Intention	5.3 (1.1)	5.6 (1.0)	0.4 (1.1)	5.2 (1.3)	6.1 (0.6)	0.9 (1.6)	0.5 (2.0)	0.36
Planning								
Sitting Self-efficacy	14.9 (4.3)	17.2 (1.9)	2.3 (4.3)	15.4 (3.8)	15.2 (3.3)	-0.2 (3.6)	-2.6 (5.8)	0.63
Planning	1.4 (0.6)	2.1 (0.8)	0.6 (1.2)	1.6 (0.9)	2.7 (0.7)	1.1 (1.2)	0.4 (1.7)	0.42
Wellbeing								
WEMWBS	50.6 (4.8)	50.6 (6.1)	0.0 (3.2)	51.0 (8.1)	53.2 (8.9)	2.2 (10.9)	2.2 (9.5)	0.27
Positive affect	35.4 (4.9)	33.4 (5.8)	-2.0 (3.6)	32.1 (7.2)	35.6 (5.7)	3.4 (9.9)	5.4 (10.7)	0.72
Negative affect	16.1 (4.9)	15.1 (5.2)	-1.0 (4.6)	16.6 (6.6)	14.6 (4.1)	-2.0 (6.6)	-1.0 (8.1)	0.18
Life satisfaction	6.4 (2.5)	6.4 (2.1)	0.0 (2.5)	6.9 (1.5)	7.3(1.7)	0.4 (2.1)	0.4 (3.8)	0.17
Worthwhileness	8.0 (1.9)	7.4 (0.7)	-0.6 (2.1)	7.4 (1.5)	7.7 (1.3)	0.2 (1.4)	0.8 (2.6)	0.45
Happiness	7.8 (2.0)	7.3 (1.6)	-0.4 (2.8)	7.2 (1.5)	7.8 (1.3)	0.6 (2.1)	1.0 (4.2)	0.40
Anxiety	3.0 (2.8)	2.6 (1.9)	-0.4 (1.6)	2.78 (2.1)	2.78 (2.3)	0.0 (1.6)	0.4 (2.5)	0.25

Note: WEMWBS—Warwick-Edinburgh Mental Well-being Scale.
