# Supplementary figures and images for: Randomised Controlled Feasibility Study of the MyHealthAvatar-Diabetes Smartphone App for Reducing Prolonged Sitting Time in Type 2 Diabetes Mellitus
Source: Int J Environ Res Public Health. 2020 Jun 19;17(12):4414. doi: 10.3390/ijerph17124414 (PMC7345154; doi:10.3390/ijerph17124414)

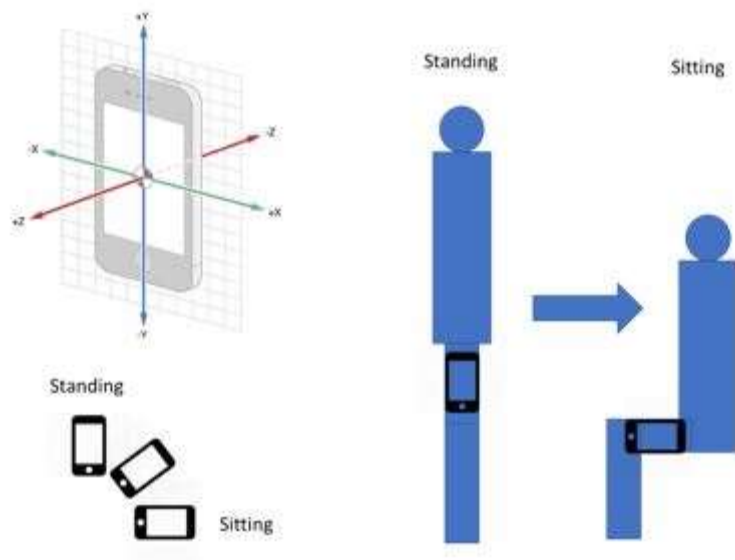

Figure S1: Sitting Detection Monitoring in the MyHealthAvatar-Diabetes app.

Supplement: Supplementary file 1 [file ijerph-17-04414-s001.zip › Supplemantary figure S1.pdf]

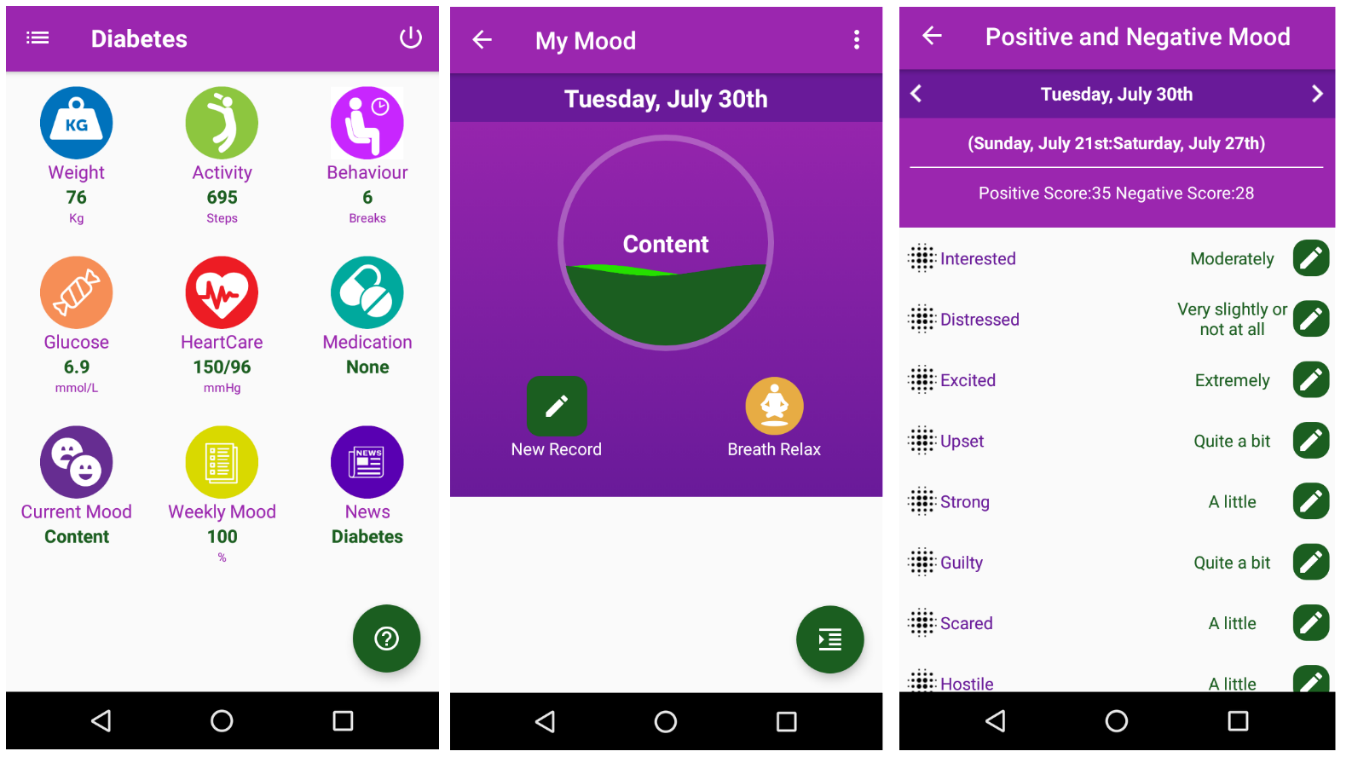


**Figure S2: MyHealthAvatar-Diabetes app health and lifestyle suites.**

Supplement: Supplementary file 1 [file ijerph-17-04414-s001.zip › Supplementary figure S2 MHS app screenshots.docx]

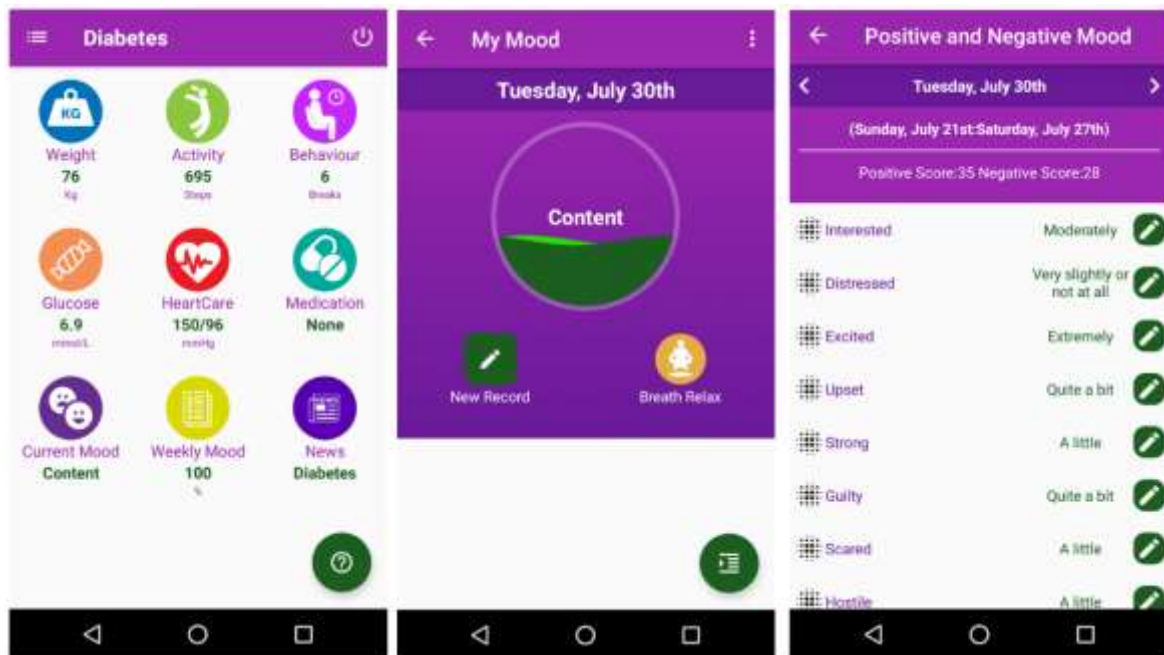

Figure S2: MyHealthAvatar-Diabetes app health and lifestyle suites.

Supplement: Supplementary file 1 [file ijerph-17-04414-s001.zip › Supplementary figure s2 MHS app screenshots.pdf]
